# Supplementary material for: THRIVEair: A community-based air monitoring network design in a pollution-burdened Philadelphia neighborhood to advance environmental justice
Source: medRxiv. 2025 Nov 23:2025.11.20.25340037. Preprint. [Version 1] doi: 10.1101/2025.11.20.25340037 (PMC12668090; doi:10.1101/2025.11.20.25340037)
Supplement: Supplement 1 [file media-1.pdf]

Supplemental Figures

Table of Contents

Figure S1. Philadelphia Energy Solutions (PES) refinery benzene data…………………………..2

Figure S2. Philly Thrive 2022 action arc 3

Figure S3. Study domain selection……………………………………………………….....4

Figure S4. THRIVEair source classes……………………………...……………………......4

Figure S5. Information tags……………...………………………….……...……………………......5

Figure S6. Benzene fact sheet……………....…………………….……...……………………......5

Figure S7. Study design fact sheet……………....……………………..……...……………………......6

Figure S8. R Shiny App Screenshots……………....……………………..……...……………………......7

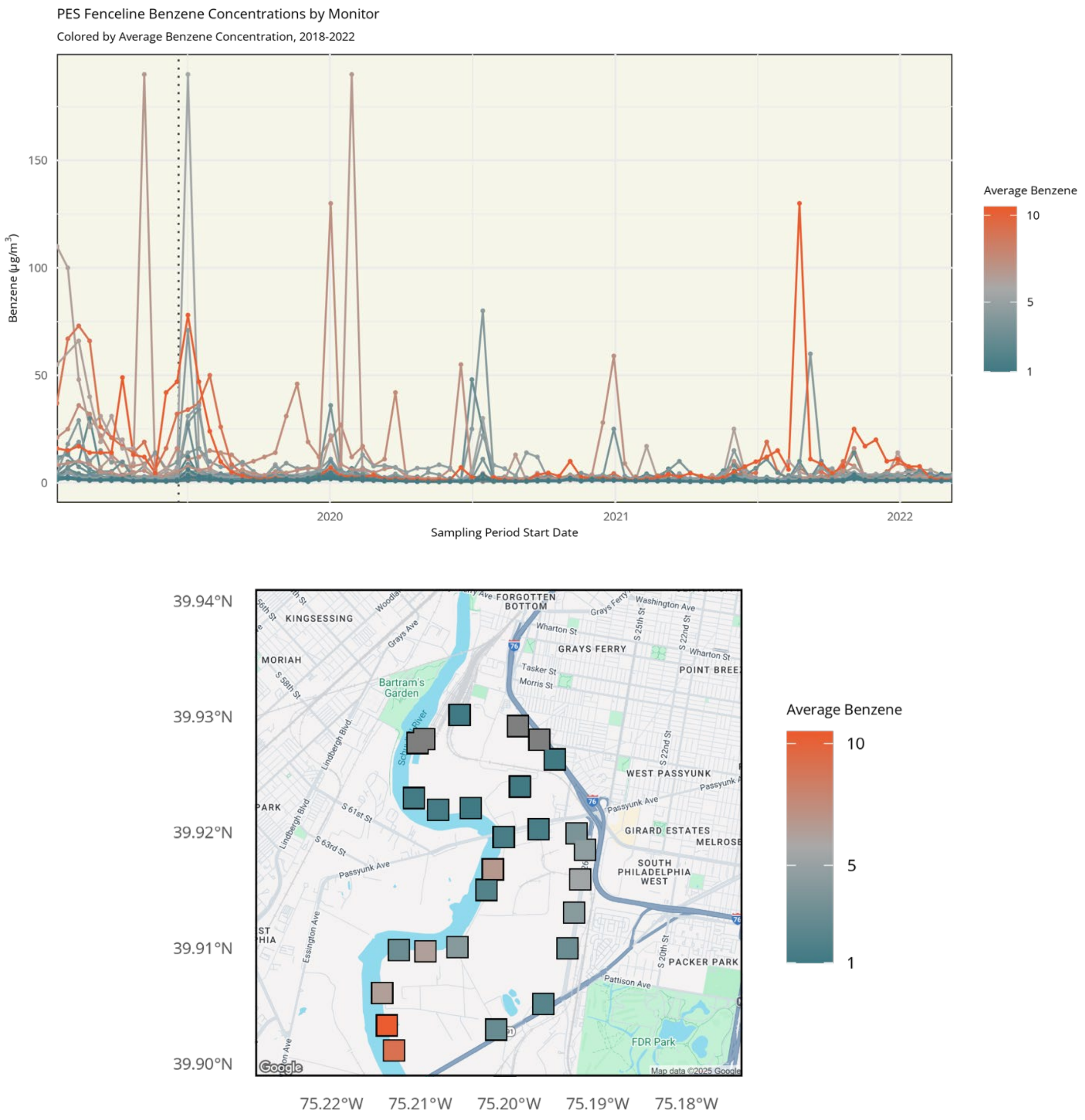

Figure S1. Philadelphia Energy Solutions (PES) refinery benzene data (2-week sampling sessions) reported to the US EPA from 2018-2022 by individual benzene monitor (top), and monitor site locations (bottom), colored by average benzene across the time period.

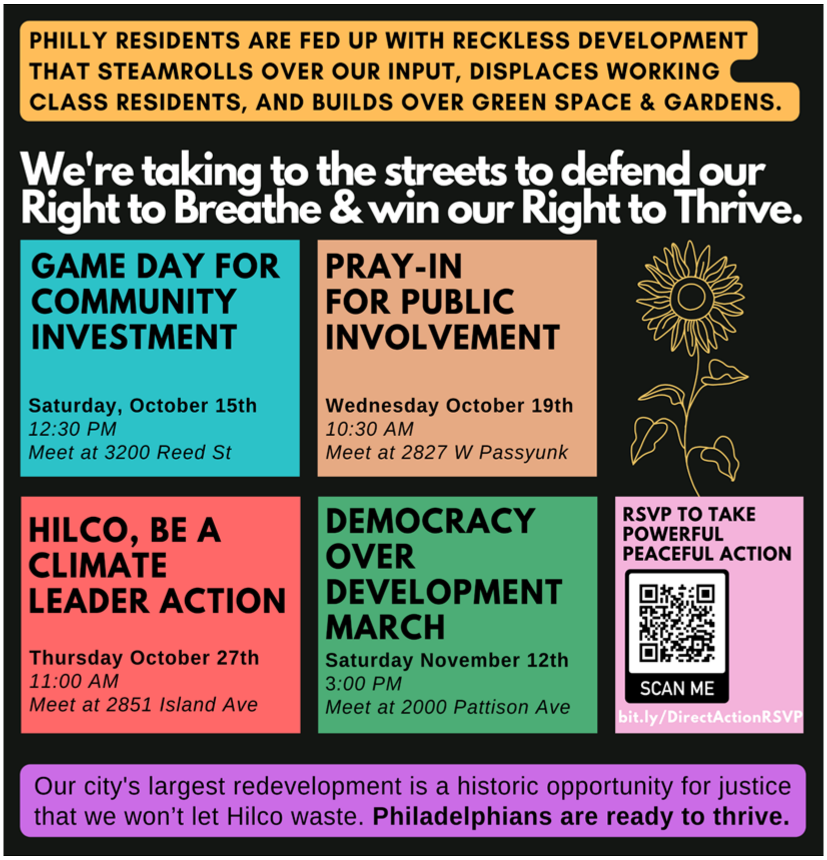

Figure S2. Philly Thrive's four-part non-violent direct action arc aimed to put pressure on HRP to negotiate a community benefits agreement through a public participation process. The third protest "Hilco be a Climate Leader" was in opposition to Philadelphia Air Management Services' announcement confirming the approval of HRP's permit to continue operations of the Schuylkill River tank farm.

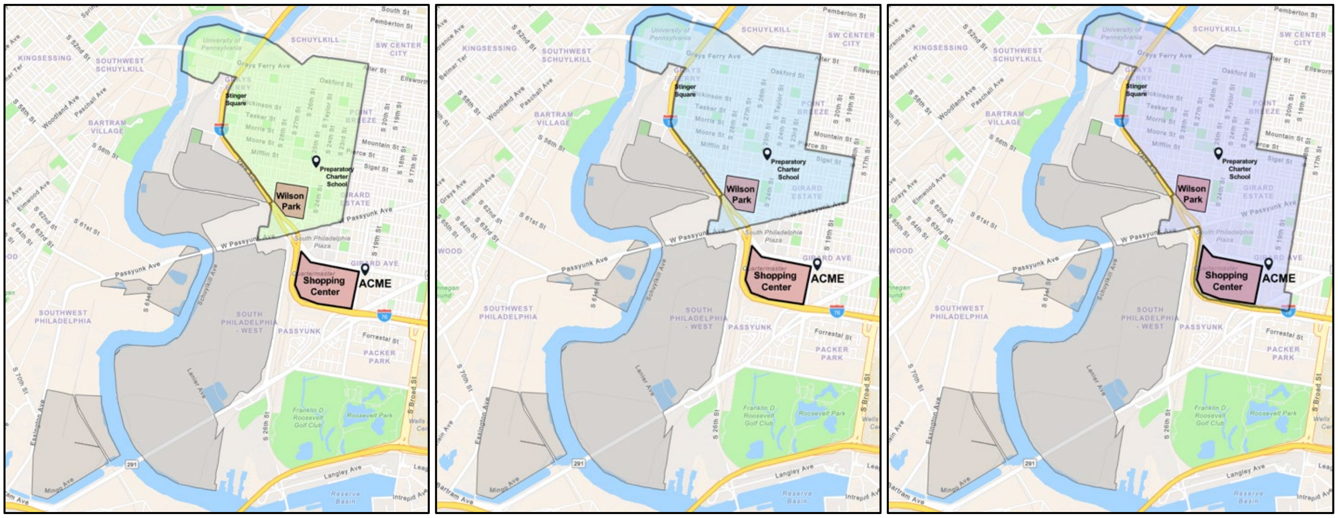

Figure S3. Study domain selection: 3 community domains presented to study design team.

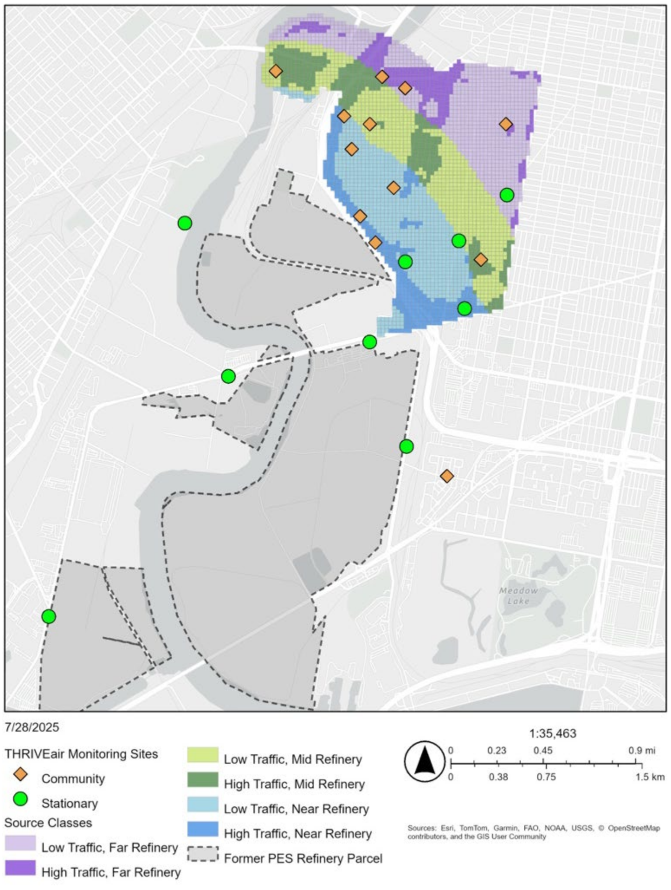

Figure S4. THRIVEair sources classes used for monitoring site selection in the community study domain.

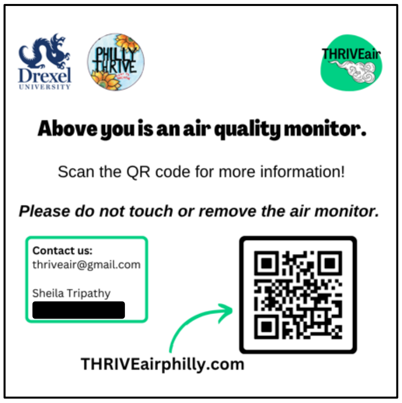

Figure S5. Information tags included underneath each air monitor. Contact phone number is redacted.

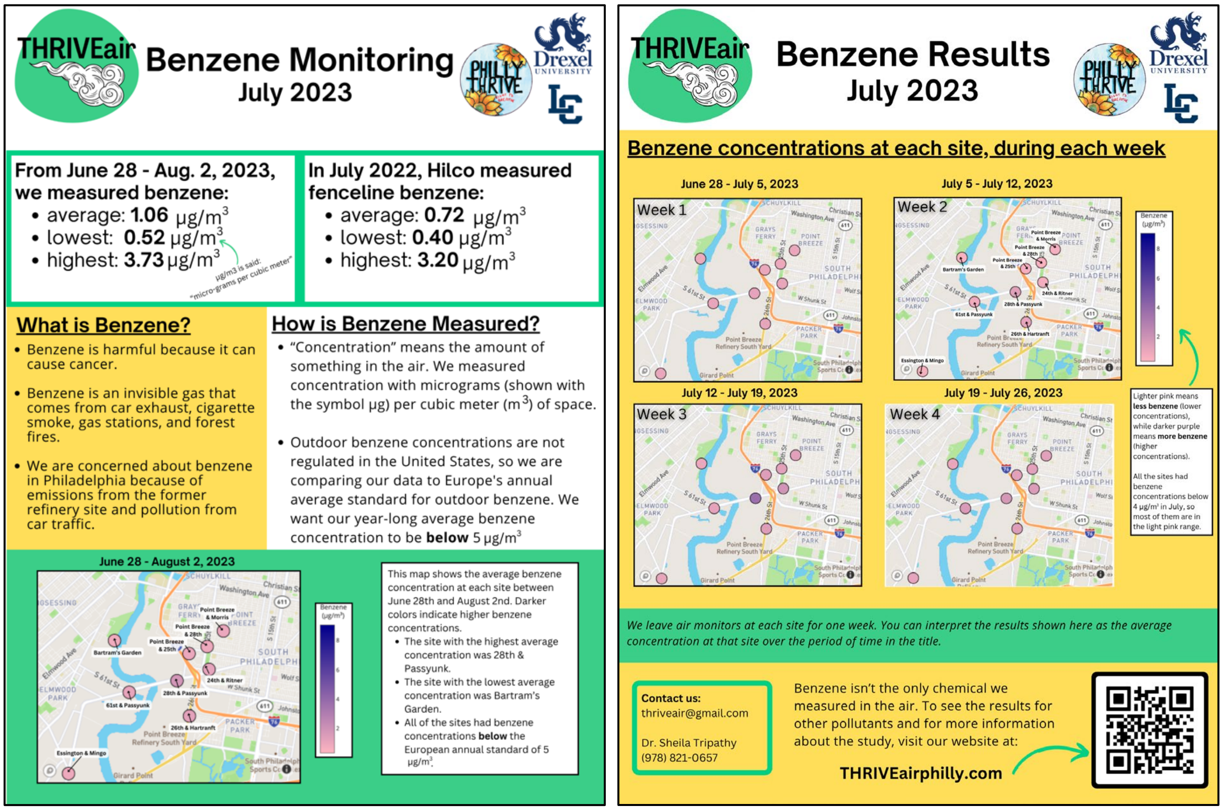

Figure S6. THRIVEair benzene fact sheet example.

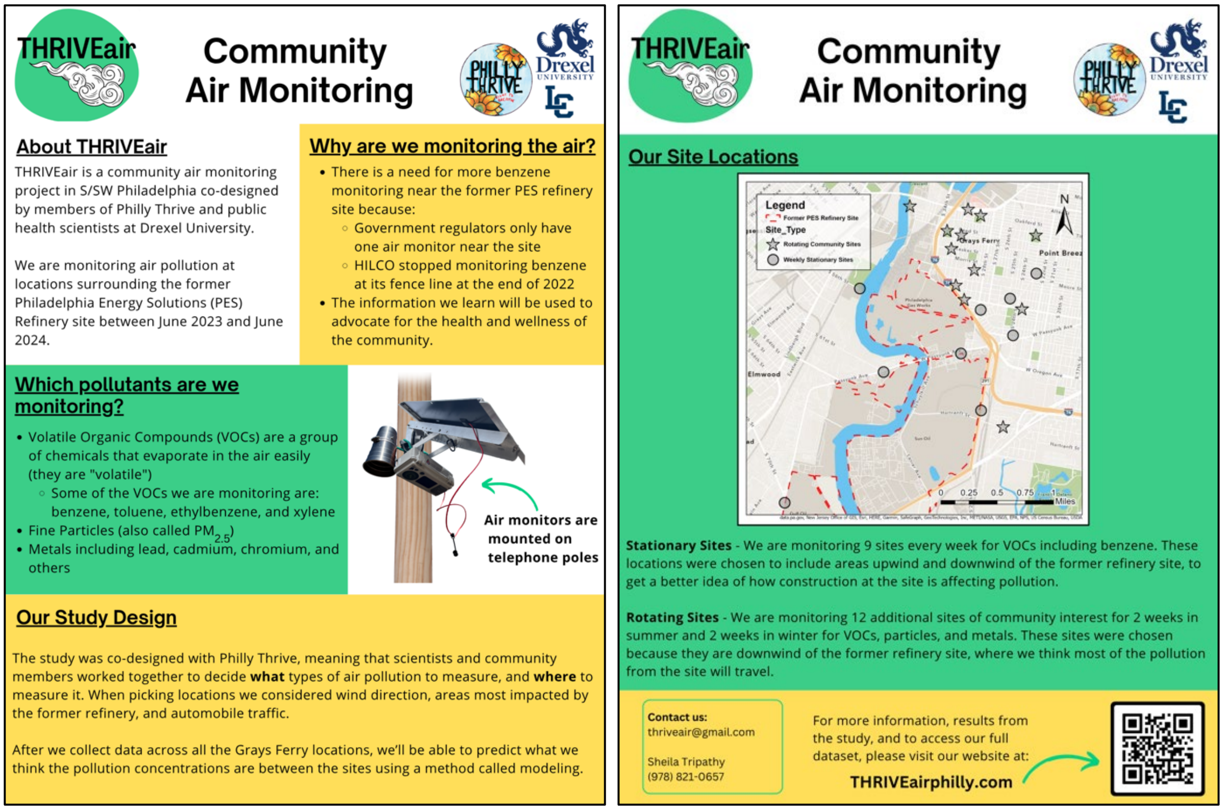

Figure S7. THRIVEair study design fact sheet.

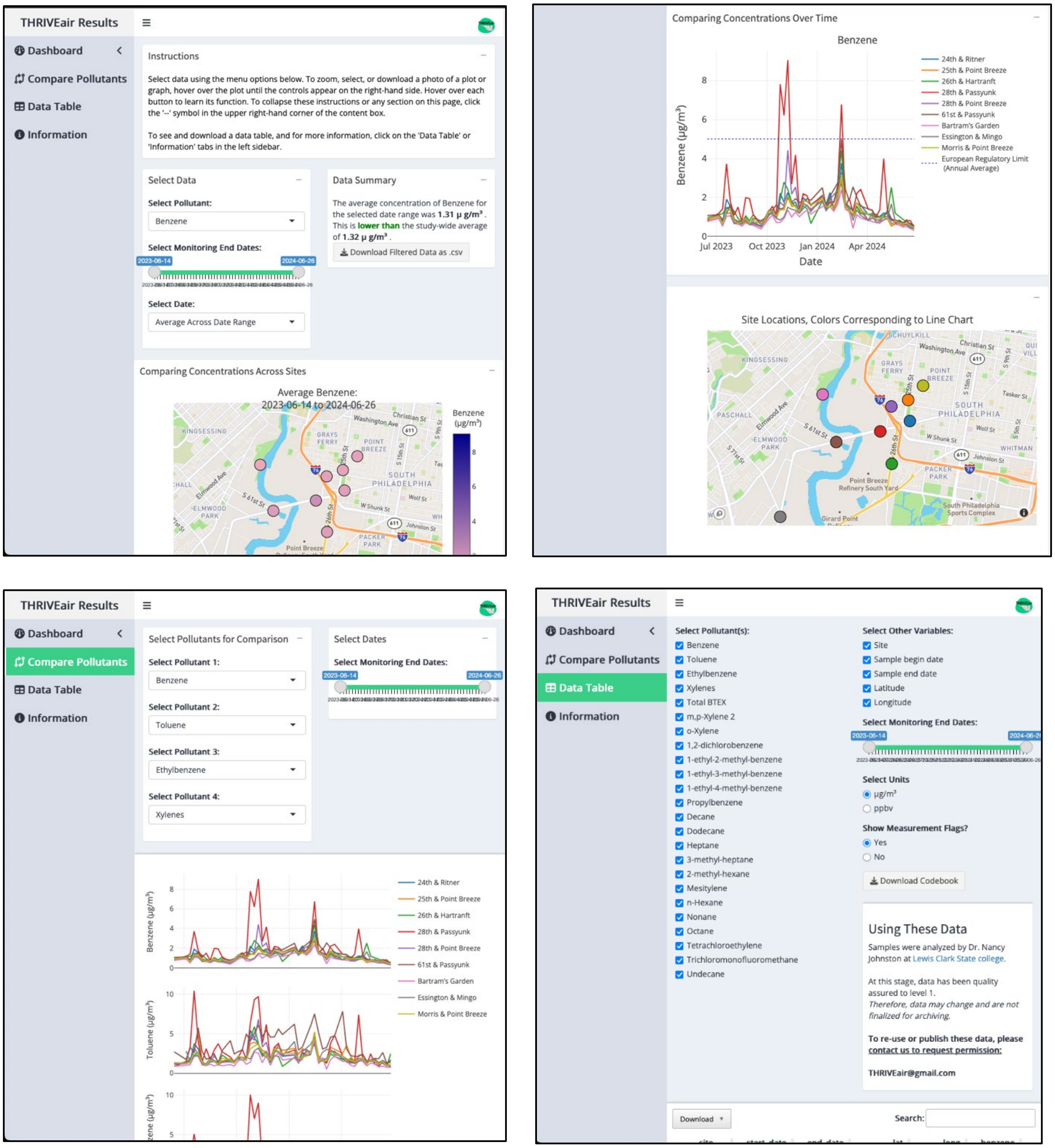

Figure S8. Screenshots from the thriveairphilly.com interactive R Shiny app.
